# Complete Genome Sequence of *Bifidobacterium actinocoloniiforme* Type Strain DSM 22766^T^, Isolated from Bumblebee Digestive Tracts

**DOI:** 10.1128/genomeA.01084-15

**Published:** 2015-09-24

**Authors:** Xiaohan Chen, Zhiguo E, Donglu Gu, Longxian Lv, Yudong Li

**Affiliations:** a Department of Bioengineering, School of Food Sciences and Biotechnology, Zhejiang Gongshang University, Hangzhou, China; b China National Rice Research Institute, Hangzhou, China; c State Key Laboratory for Diagnosis and Treatment of Infectious Disease, The First Affiliated Hospital, Zhejiang University, Hangzhou, China

## Abstract

Bifidobacteria are one of the most important beneficial bacteria in the gut of mammals and insects. We sequenced the genome of *B. actinocoloniiforme* DSM 22766, which was isolated from the digestive tracts of bumblebees. The genome contains 1,548 protein-coding genes, 49 RNAs and two CRISPR repeats.

## GENOME ANNOUNCEMENT

Bifidobacteria, belonging to the class *Actinobacteria*, are the main representatives of animal and human probiotics (1). The type strain *Bifidobacterium actinocoloniiforme* DSM 22766 (CCM 7728) was isolated from the digestive tracts of bumblebees (2). Bumblebees are the second most economically valuable pollinators that are surpassed only by honey bees. However, the populations of bumblebees have now been declining, which are primarily driven by infectious diseases (3). The gut microbiota is critical to the health of bumblebees, especially for defense against pathogens and parasites (4).

The genome of *B. actinocoloniiforme* DSM 22766 was sequenced with the next-generation sequencing platforms Illumina MiSeq and HiSeq 2000. The paired-end libraries (550-bp insert size) were generated according to the manufacturer’s instructions. The total number of reads (300 bp) based on MiSeq sequencing is 2 × 4,179,156, and the number of reads (100 bp) based on HiSeq 2000 is 2 × 4,957,629 bp. The draft genome sequence was assembled using SPAdes version 3.50 and the A5-miseq pipeline (5). The number of the contigs is 18, of which the *N*_50_ is 186.2 Kb, and the max length was 833.5 Kb. Both assemblies were mapped to the reference genome sequence (NCBI accession number JGK01) using CLC Genomics Workbench (CLC Bio, Aarbus, Denmark). Gap closing was performed by GapFiller (6) and multiplex PCR. The gene prediction and annotations of the genome sequence of *B. actinocoloniiforme* DSM 22766 were performed using the RAST (Rapid Annotation using Subsystem Technology) server (7). Transfer RNA (tRNA) genes were detected using tRNAScan-SE. Ribosomal RNAs (rRNAs) were identified using a BLASTn search against the rRNA databases. The clustered regularly interspaced short palindromic repeat (CRISPR) repeats was analyzed using the CRISPRfinder (http://crispr.u-psud.fr) (8).

The genome consists of one chromosome and one plasmid. The size of the genome sequence is 1,830,060 bp, and the GC content is 62.71%. These sequences contained 1,548 protein-coding genes, 47 tRNAs, and 2 complete 5S-23S-16S rRNA gene clusters. The majority of the protein-coding genes were annotated with a putative function (about 1,172 protein-coding genes). *B. actinocoloniiforme* contain the gene coding for fructose-6-phosphate phosphoketolase (F6PPK), the key enzyme of hexose catabolism in members of the genus *Bifidobacterium*, through which hexoses are degraded to acetic and lactic acids. Compared with honey bee–associated *Bifidobacterium* strains (9), strain DSM 22766 also possesses genes to cope with reactive oxygen species, including catalase, peroxidase, and superoxide-dismutase, which help protect against the negative effects produced by oxygen in an aerobic environment. Osmotic pressure change within the gut has been suggested as a main digestive mechanism capable of breaking pollen cells (10). Consistent with adapting to harsh osmoregulatory conditions, strain DSM 22766 contains the transmembrane channel aquaporin Z, which is highly stable and facilitates both rapid and long-term osmoregulation. Two CRISPR repeats and 7 CRISPR-associated proteins were detected to provide immunity against genetic parasites. Comparative genomic analysis of different strains may provide candidate strains for probiotic treatment applied widely in the food industry.

### Nucleotide sequence accession number.

The complete genome sequence and annotation of *Bifidobacterium actinocoloniiforme* DSM 22766 strain were deposited in DDBJ/EMBL/GenBank under the accession number CP011786.
