# Erratum for Chen et al., Complete Genome Sequence of *Bifidobacterium actinocoloniiforme* Type Strain DSM 22766^T^, Isolated from Bumblebee Digestive Tracts

**DOI:** 10.1128/genomeA.01349-15

**Published:** 2015-10-22

**Authors:** Xiaohan Chen, Zhiguo E, Donglu Gu, Longxian Lv, Yudong Li

**Affiliations:** a Department of Bioengineering, School of Food Sciences and Biotechnology, Zhejiang Gongshang University, Hangzhou, China; b China National Rice Research Institute, Hangzhou, China; c State Key Laboratory for Diagnosis and Treatment of Infectious Disease, The First Affiliated Hospital, Zhejiang University, Hangzhou, China

## ERRATUM

Volume 3, no. 5, e01084-15, 2015. Page 1: The byline and affiliation line should read as given above.

